# 

**Published:** 1899-08

**Authors:** 


					AUGUST, 1899.
THE
AM E R1 ^ A Nj J y Vi>;?? -
OF
DENTAL -SeiENCEL
EDITED BY
F. J. S. GORGAS, M. D., D. D. S.
RICHARD GRADY, M. D, D. D. S.
Vol. 33.
THIRD SERIES
ffo. 4.
BALTI MORE:
SNOWDEN & COWMAN MFG. CO., 9 W. Fayette St.
LONDON:
TRUBNER 4, CO., 60 Paternoster Row.
Entered at the Post Office at Baltimore, Md., as Second-class Matter.
PH.YHBLE IN HDVHNCE.
[PUBLISHED MONTHLY.
$2.50 PER SNNUM.I

				

